# Pelvic Floor Muscle Anatomy and its Contribution to Penile Erection in Olive Baboons

**DOI:** 10.5152/tud.2024.23020

**Published:** 2024-05-01

**Authors:** Roman Ovchinnikov, Ilya Pyatnitskiy

**Affiliations:** 1 Department of Physiology, Pirogov Russian National Research Medical University, Moscow, Russia; 2 Department of Biomedical Engineering, The University of Texas at Austin Cockrell School of Engineering, Austin, USA

**Keywords:** Erectile dysfunction, ischiocavernosus muscle, pelvic floor, penile erection, penis, perineum

## Abstract

**Objective:**

Despite the number of studies on the contribution of pelvic floor muscles (PFM) to the penile erection process, their significance is still underestimated. The goal of this study was to investigate the role of PFM in the erection process in non-human primates.

**Materials and Methods:**

First, we performed an anatomical study of the penile structures in 12 baboon cadavers. Next, we created chronic electrophysiological models of normal erectile function in 25 olive baboons. We implanted electrodes on the cavernous nerves to control penile blood filling and placed other electrodes on the pudendal nerve to stimulate the ischiocavernosus muscles (ICM) contractions after sufficient blood filling, thus simulating both vascular and muscular phases of penile erection. We controlled the intracavernous pressure (ICP) during nerve stimulations and further performed a mathematical analysis of the collected data.

**Results:**

We described the main pro-erectile muscle anatomy and its relationship with penile structures in monkeys. During neurostimulation studies, we showed the key role of ICM in achieving full penile rigidity with suprasystolic ICP up to 120-300 mm Hg. We also developed a math model for calculating the pro-erectile muscle’s contraction force with high accuracy.

**Conclusion:**

In this study, we demonstrated the crucial role of PFM in the penile erection process using the monkey model, supporting the previous studies’ data. We consider these results essential for a better, more complex understanding of the penile erection process and the participating structures in each stage. This is essential for further improving and designing novel erectile dysfunction diagnostics and treatment techniques.

## Introduction

Penile erection is a complex process consisting of several stages with the participation of different systems. Erection represents the final common pathway of the integrative synchronized action of psychological, neuronal, hormonal, vascular, and cavernous smooth muscle systems.^1,2^

The male pelvic floor, which includes the ischiocavernosus muscles (ICM) and the bulbospongiosus muscle (BSM), serves 3 primary roles: providing support for the pelvic organs, preventing incontinence by promoting the voluntary closure of the urethral and anal sphincters, and facilitating erectile and ejaculatory processes.^3,4^ Dysfunction or injury to the pelvic floor may lead to debilitating disorders, including pelvic organ prolapse, defecatory dysfunction, urinary incontinence, and sexual dysfunction.^5^

It has been demonstrated that the voluntary contraction of ICM and BSM significantly increases intracavernous pressure (ICP).^3,6^ Lavoisier et al found that ICM electromyography (EMG) bursts occur in phase with the increase in ICP, and the duration of the EMG bursts precisely covaries with the duration of phasic ICP increases.^6^ These contractions are thought to be responsible for penile rigidity, as defined by the level of ICP.^6,7^

Despite the number of studies on ICM and BSM’s contribution to the penile erection process^3,4,6-9^ and pelvic floor muscle training (PFMT) proven efficacy, it still remains under-recognized and underutilized for males.^3,4,8^ Pelvic floor muscle training is crucial for male genitourinary health, yet it lacks the recognition it deserves compared to its female counterpart.^8^ Its benefits include safety, noninvasiveness, and the potential to empower men. Currently, PFMT education in urology practice is typically limited to verbal instructions, pamphlets, or in-office sessions. However, there’s a growing demand for specialized urology services, utilizing nonphysician providers, to deliver PFMT and easily accessible PFMT programs for home use.^8^

Two nerves control the penile erection process: cavernous nerves (parasympathetic and sympathetic divisions of the autonomic nervous system) control the vascular phase, while the pudendal nerve (somatic nervous system) controls the muscular phase.^10,11^ Effective and appropriate neurostimulation of these nerves can closely mimic the physiological mechanisms underlying a normal erection.^12-14^ Cavernous nerves neurostimulation prompts nitric oxide release, causing penile smooth muscle relaxation and vasodilation, crucial for increased blood flow into the corpora cavernosa, necessary for erection. In contrast, pudendal nerves neurostimulation contracts pelvic floor muscles, compressing penile veins and aiding in erection maintenance.^12-14^

In previous studies, the pig-tail and rhesus monkey models have proven reliable for investigating penile erection due to their similarity to human penile anatomy and physiology, making them valuable tools for understanding the erection process.^15,16^ In our study, we chose to investigate the PFM role in the erection process using olive baboons *(Papio anubis)* because of their larger size, more extensive pelvic floor structures and nerves—better suited for human electrodes and pulse generator implantation—and even closer proximity to human anatomy.

First, we conducted a comprehensive examination of PFM anatomy and its anatomical relationship with penile structures in baboon cadavers. Subsequently, we established chronic electrophysiological models to mimic normal erectile function in monkeys. This involved implanting electrodes on the cavernous nerves to regulate penile blood filling and placed other electrodes on the pudendal nerve to stimulate ICM and BSM, thereby simulating both the vascular and muscular phases of penile erection. This choice of model was deliberate, as chronic electrophysiological models provide a sustained platform for studying physiological processes over time, offering insights into long-term erectile function and responses that may not be achievable with acute models.

We consider these data and results crucial for enhancing our understanding of the penile erection process and the involvement of participating structures at each stage. This comprehensive understanding is essential for further refining and developing novel diagnostic and treatment techniques for erectile dysfunction. This includes exploring electrical, optogenetic, and other neurostimulation methods, as well as expanding the implementation of PFM in patients with erectile dysfunction.

## Material and Methods

The study was conducted at the Primate Research Center. Ethical approval for the study was obtained from the Primate Research Center Ethics Committee (approval no. 45/9-17P, dated September 25, 2017).

### Anatomical Study

Anatomical investigations were conducted on 12 male olive baboon (*P. anubis*) cadavers, all of which had succumbed to natural causes and had an average age of 32 ± 4.4 years. Autopsies were carried out by a certified pathologist at a specialized facility. Furthermore, magnetic resonance imaging (MRI) scans using a Bruker 7 Tesla preclinical MRI scanner were performed on penile and pelvic floor muscle (PFM) tissues from 3 monkeys to examine PFM anatomy and its correlation with penile structures.

### Electrophysiological Experiments

**Supplementary Figure 1.**
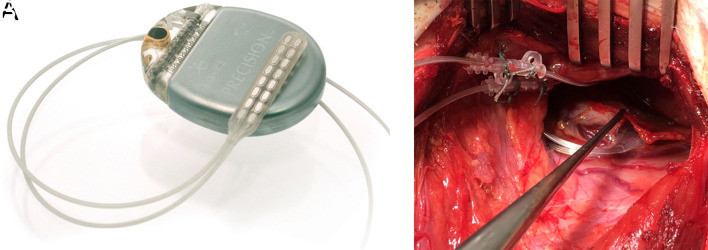
Nerve electrostimulation equipment and implantation process. (A) Flat electrode and implantable pulse generator; (B) Flat electrodes implanted on the cavernous nerves.

Electrophysiological experiments were conducted on 25 olive baboons (*P. anubis*) aged 7.3 ± 1.8 years and weighing 24.9 ± 2.3 kg. The baboons were anesthetized and implanted with cylindrical electrodes for pudendal nerve stimulation and flat electrodes for cavernous nerve stimulation (Supplementary Figure 1).

### Surgery

The induction anesthesia and additional sedation and analgesia involved a combination of Zoletil (tiletamine hydrochloride and zolazepam hydrochloride) and Xyla (xylazine hydrochloride), administered intramuscularly at average therapeutic doses based on each animal’s weight (Zoletil 0.8-1.1 mL, Xyla 2.0-2.2 mL). Intraoperatively, sedation was maintained through a continuous intravenous infusion of Propofol at 80-100 mg/hour. Throughout the surgery, the monkeys remained under anesthesia with spontaneous breathing. Anesthesia adequacy, gas exchange, and hemodynamic parameters were evaluated using general clinical criteria and cardiac monitor readings, including 3-lead ECG, BP, SpO2, RR, and thermometry.

Intracavernous pressure (ICP) was measured using an Arteriofix catheter (B. Braun Medical Inc.) inserted into the corpus cavernosum, with pressures recorded using a Philips IntelliVue MP50 anesthetic monitor (Philips, Netherlands).

A lower midline laparotomy was performed to gain surgical access to the tissues in the prostate gland’s projection, done retroperitoneally. The parasympathetic portion of the cavernous nerves was verified using a lab-designed electroprobe and stimulation mapping under ICP control (ICP increase). The flat electrode was oriented parallel to the neurovascular bundle and fixed to the perineurium and surrounding tissue using Prolene 8/0 thread (Supplementary Figure 1B). An external generator trial supply of energy was then performed to assess the electrodes’ optimal positioning, including registration of the stimulation threshold, measurement of impedance, and exclusion of parasitic stimulation. The wire part of the electrodes was anchored to the wound and to the aponeurosis of the anterior abdominal wall muscles, accounting for the margin of length to avoid traction during pelvic organ displacement.

**Supplementary Figure 2.**
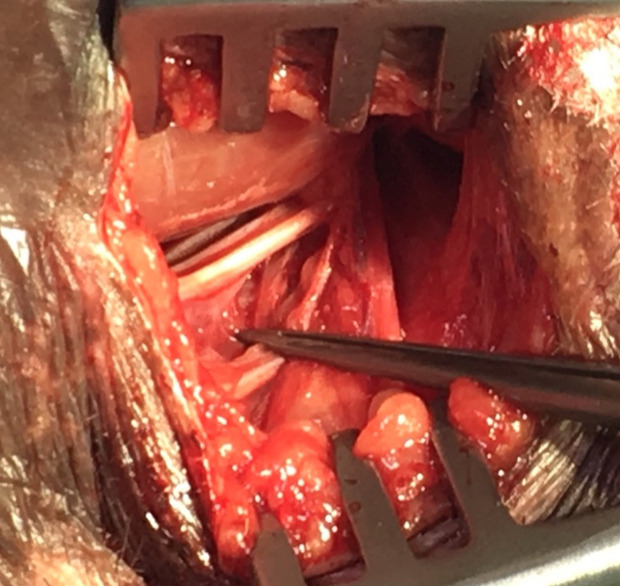
Pudendal nerve isolation (arrow) for further implantation of cylindrical electrodes.

The electrodes for pudendal nerve stimulation were implanted through a linear incision 2.5-3 cm long, approximately 1.8-2.2 cm from the anus. A neurovascular bundle was isolated at a depth of 2-3 cm from the wound edges (Supplementary Figure 2). The first pudendal nerve was identified using electrostimulation with an electric probe, resulting in ICM contraction. A cylindrical electrode was positioned and fixed parallel to the nerve, and the same implantation was done on the opposite side. Electrodes were sutured to the perineurium or nearby tissues using a similar method, and specialized anchors were used as wire clamps. A loop was placed in the wound to compensate for tension during body movement.

After electrode implantation, wires from the pudendal nerve electrodes were passed through the pelvic diaphragm into the pelvis and fixed with an anchor to the anterior abdominal wall. Subsequently, a pulse generator was implanted under the rectus abdominis muscle’s aponeurosis in the formed pocket and connected by the connector part with the wires from the pudendal nerve and cavernous nerves electrodes.

## Results

### Anatomical Study

**Figure 1.**
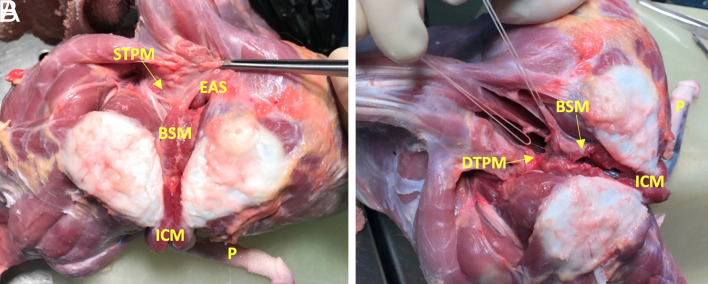
Pelvic floor muscles of the male olive baboons. (A) Bottom view; (B) Side view. ICM, ischiocavernosus muscle; BSM, bulbospongiosus muscle; DTPM, deep transverse perineal muscle; STPM, superficial transverse perineal muscle; EAS, external anal sphincter; P, penis.

**Figure 2.**
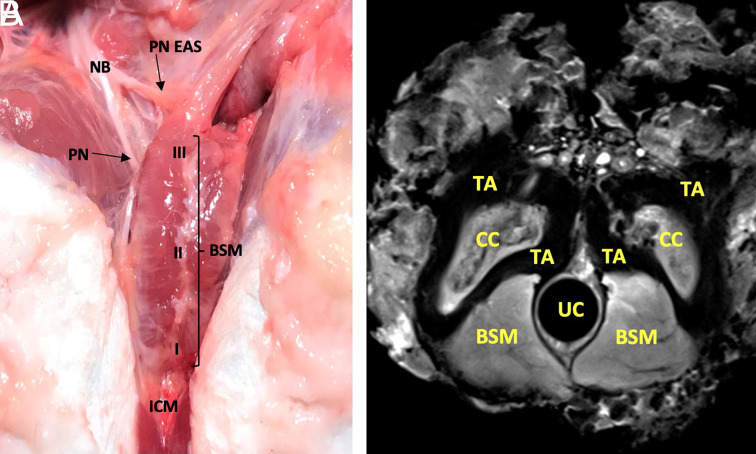
Anatomical section, posterior view (A), and cadaveric tissue 7T MRI (B) of bulbospongiosus muscle and surrounding structures. BSM, bulbospongiosus muscle (I, anterior part; II, middle part; III, posterior part); ICM, ischiocavernosus muscle; NB, neurovascular bundle; PN, Branches of the pudendal nerve to the external anal sphincter; UC, urinary catheter; CC, corpus cavernosum; TA, tunica albuginea.

**Figure 3.**
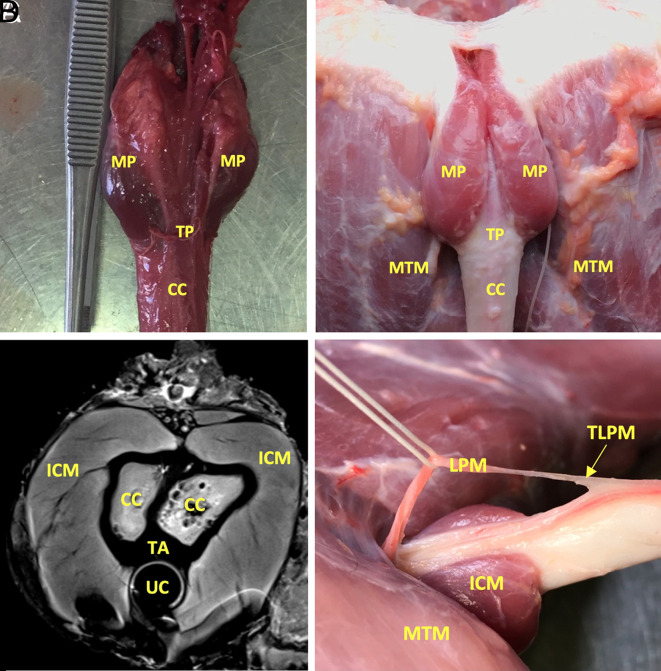
Anatomical section (A, upper view and B, bottom view), and cadaveric tissue 7T MRI (C) of ischiocavernosus muscle (ICM) and surrounding structures. Anatomy of levator penis muscle (D). TP, ICM tendon part; MP, ICM muscle fibers part; MTM, medial thigh muscles; CC, corpus cavernosum; TA, tunica albuginea; UC, urinary catheter; LPM, levator penis muscle; TLPM, tendon of the levator penis muscle.

In functional terms, 3 muscles directly contribute to the erection process in monkeys: the ischiocavernosus muscle (ICM), bulbospongiosus muscle (BSM), and levator penis muscle (LPM) (Figures 1-3 and Tables 1-3). These muscles are situated in the urogenital triangle of the perineum and are connected to the penis. The base of the urogenital triangle is formed by the superficial and deep transverse muscles of the perineum.

### Bulbospongiosus Muscle

The bulbospongiosus muscle (BSM) is a bilateral muscle with a plate-like form, measuring 8.9 ± 1.1 mm thick, which tightly covers the bulb and extends to a part of the corpus spongiosum (CS). The posterior scrotal artery, vein, and nerves traverse along the lateral surface of the muscle in an oblique direction. The muscle comprises 3 parts: anterior, middle, and posterior (Figure 2A).

Bulbospongiosus muscle functions: BSM contraction contributes to the compression of the CS bulb and the extrusion of sperm (during ejaculation) or the last drops of urine (during urination). Additionally, when the middle bundles contract, the deep and urethral veins are compressed, hindering venous return and increasing pressure in the glans and corpus cavernosum.

The anterior part of the BSM originates from the median suture of the tunica albuginea (TA) of the CS in the anterior region of the bulb over a length of 22.7 ± 2.3 mm (Figure 1). The muscle fibers have an oblique direction and extend onto the lateral surface of the corpus cavernosum (CC) from the CS, where they are partially interwoven into the TA. Upon reaching the dorsum of the CC, they entirely merge into the tendons and firmly attach to the TA. The length of the muscle fibers is 3.8 ± 0.7 cm. The posterior bundles of the BSM’s tendons are interconnected with the lateral bundles of the ischiocavernosus muscle (ICM). These interconnected tendons from both muscles on opposite sides form a tendon loop, measuring 9.1 ± 0.9 mm wide, on the dorsum of the penis, providing robust support for the attached muscles and securing the anterior part of the bulb to the CC. The neurovascular bundle penetrates under the muscle from the side and behind, passing to the muscle and the bulb of the CS. Considering the direction of the muscle fibers, the anterior part of the BSM can also compress the CS from the sides, promoting blood flow from the bulb into the distal part of the CS, particularly into the glans penis.

The middle part of the muscle is the largest. Its attachment line to the median suture in the bulb area measures 2.8 ± 0.7 cm in length. The muscle fibers are directed anteriorly in an oblique manner, covering the lateral surfaces of the bulb and intertwining with the TA of the CC, forming short tendons before attachment. The length of the fibers is 2.7 ± 0.3 cm. The contraction of the middle bundles results in compression of the deep and urethral veins, hindering venous return and increasing pressure in the glans and CC.

The posterior part of the BSM is the shortest, originating from the central tendon of the perineum and interwoven into the posterior part of the bulb along the midline. Its fibers exhibit an almost transverse orientation and are reinforced by fibers of the deep transverse perineal muscle and the external anal sphincter (Figure 1). The posterior bundles contribute to the fixation of the bulb of the penis and its displacement toward the center of the perineum.

### Ischiocavernosus Muscles

Ischiocavernosus Muscles (ICM) are bilateral and elongated, with a muscular origin and tendon attachment (Figure 3). The muscle originates behind the CC crura from the ischial tuberosity, the ramus of the ischium, and the sacrotuberous ligament. It covers the crura and the lower surface of the CC, intertwining with the TA of the CC. Approximately midway along its length, the ICM transitions into a tendon, although this transition point exhibits significant variability. The muscle comprises longitudinally oriented fiber bundles that envelop the CC crura on 3 sides (Figure 3C). The length of the muscle fibers is 4.5 ± 0.4 cm, with the location of the fibers distinguishing the lateral, middle, and medial parts.

### ICM Functions

The ICM presses the CC crura against the pubic symphysis and provides tension to the TA, displacing its surface layer, thus preventing blood outflow from the crura and contributing to CC blood filling and penile erection.

The lateral part of the muscle is attached to the lateral and dorsal surfaces of the CC. Some tendon bundles connect with the tendons of the anterior part of the BSM and contribute to the formation of the tendon loop described earlier on the dorsal surface of the penis. The tendon of the lateral part of the ICM attaches to the periosteum of the pubic bone, forming a well-defined tendon bundle measuring 8.1 ± 1 mm in width and 2 ± 0.3 mm in thickness. A broader bundle (width 13.5 ± 1.3 mm) is attached to the area of the symphysis. Based on the orientation of the fibers, the function of the lateral part of the ICM is to press the CC crura against the pubic symphysis.

The middle part of the muscle comprises the longest muscle fibers, with their transition to tendon occurring only at the end. These fibers are interwoven into the lateral surfaces of the CC and cover 2.3 ± 0.3 cm of the outer aspect of the penis, forming easily visible subcutaneous beads at the base of the penis. During erection, this part of the muscle provides tension to the TA and displacement of its surface layer.

Short tendons attach the medial part of the ICM to the lower surface of the CC in the region of the urethral groove. These tendons are connected to the TA and the septum of the penis and are intertwined with the corresponding tendons on the opposite side. Additionally, they are interwoven into the thin tunica albuginea of the anterior part of the bulb and the proximal CS. Consequently, the lower surface of the CC and the CS in the region of the urethral sulcus adhere firmly to each other. The medial part contributes to the stabilization of the cavernous-spongy contact in the area of the urethral groove.

### Levator Penis Muscle

The Levator Penis Muscle (LPM) is bilateral and elongated (Figure 3D).^17^ It originates from the periosteum of the pubic bone and is woven by the tendon part into the suspensory ligament. The muscle fibers are 3.8 ± 1.1 cm long and 2.3 ± 0.5 mm in diameter, with a longitudinal orientation. It transitions into a tendon cord, measuring up to 15 ± 0.5 cm long and 0.7 ± 0.2 mm in diameter, which merges with Buck’s fascia at the middle third level of the penis. Often, at this level, macro-anatomically, the tendons on the right and left sides fuse into a single cord. The distal attachment of the tendon occurs on the dorsal plane of the baculum.

In our view, the term “levator penis muscle” does not entirely reflect its functional characteristics. Based on anatomical findings, this muscle displaces the skin and foreskin in the proximal direction during an erection and contributes to the curvature of the flexor side of the glans penis. Therefore, we propose it be more appropriately named the “Glans penis flexor muscle.”

### Electrophysiology Experiments

At least 2 weeks after the primary surgery, monkeys with an implanted generator and electrodes on the cavernous nerves and pudendal nerve were studied in the electrophysiological operating room under anesthesia.

We used the following physiological parameters of stimulation: a rectangular monopolar pulse with a duration of 1000 μs, a frequency of 100 Hz, and an amplitude of 4.1 ± 0.8 mA. The ICM and BSM contraction parameters and effects on the cavernous tissue were evaluated. The depth and adequacy of anesthesia, blood pressure, ICP, linear dimensions, and cross-sectional area (according to ultrasound data) of the penis’s outer part were monitored during cavernous nerves and pudendal nerve electrostimulation. ICM and BSM contractions were monitored electromyographically using bipolar intramuscularly inserted needle electrodes.

**Figure 4.**
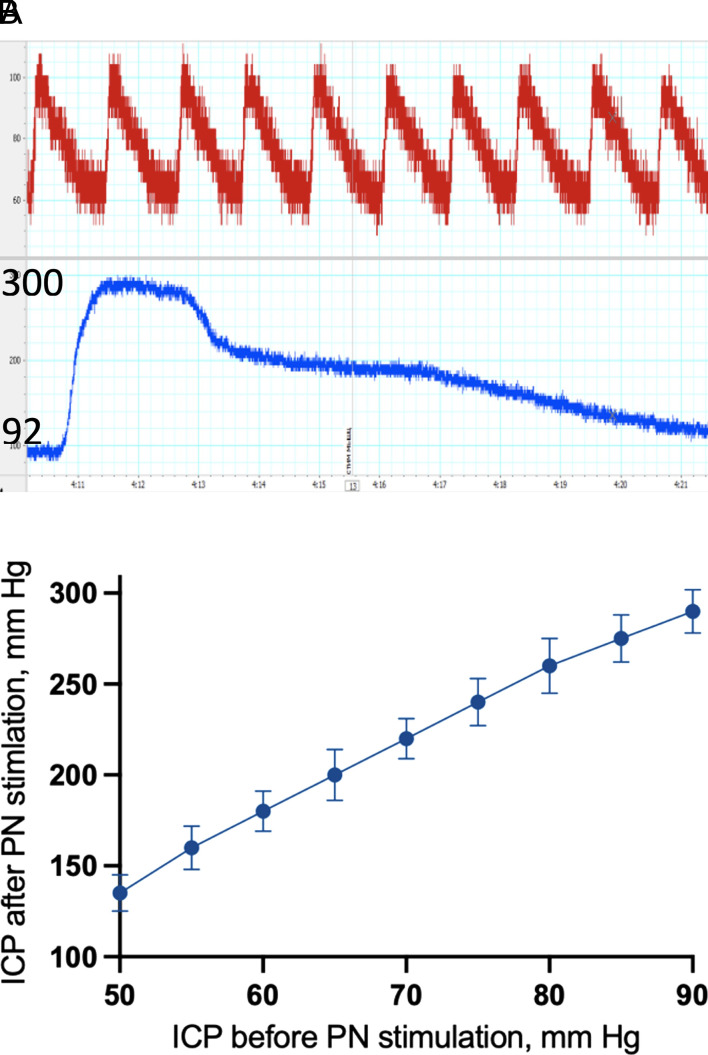
(A) Intracavernous pressure (ICP) changes in response to electrical stimulation of the pudendal nerve (blue curve) in Monkey #1. The red curve represents the invasive arterial pressure recorded in the femoral artery. This control was mandatory to exclude the influence of fluctuations in systemic hemodynamics on penile blood flow. Pressure before pudendal nerve stimulation = 92 mm Hg, and during pudendal nerve stimulation = 300 mm Hg. E = 0.138 J; F = 7.19 N. (B) Graph of the maximum ICP values (±SD) achieved due to pudendal nerve stimulation, depending on the initial ICP value (after cavernous nerve stimulation) (n = 25).

**Supplementary Figure 3.**
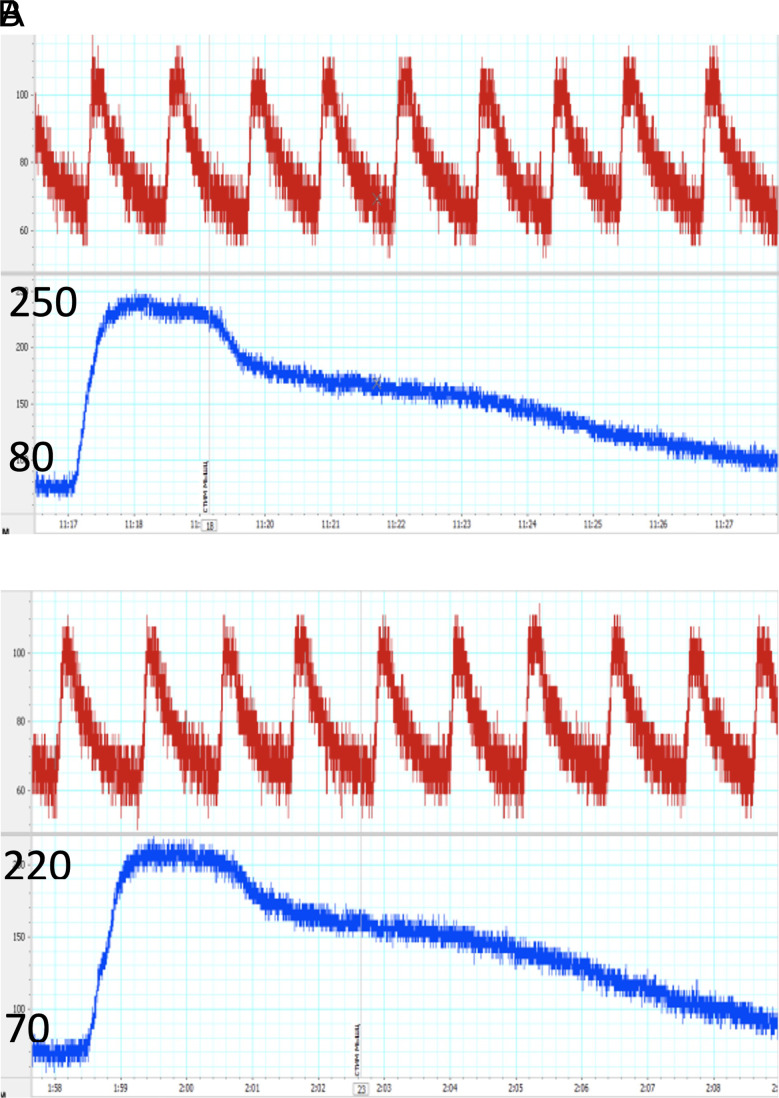
Intracavernous pressure (ICP) changes in response to electrical stimulation of the pudendal nerve (blue curve). The red curve represents the invasive arterial pressure recorded in the femoral artery. This control was mandatory to exclude the influence of fluctuations in systemic hemodynamics on penile blood flow. (A) Monkey #2. Pressure before pudendal nerve stimulation = 80 mm Hg, and during pudendal nerve stimulation = 250 mm Hg. E = 0.113 J; F = 5.88 N. (B) Monkey #3. Pressure before pudendal nerve stimulation = 70 mm Hg, and during pudendal nerve stimulation = 220 mm Hg. E = 0.10 J; F = 5.19 N.

In the first stage, we stimulated cavernous nerves under ICP control, achieving significant penile blood filling and an ICP increase from 15 ± 3 mm Hg up to 72 ± 23 mm Hg (Figures 4 and Supplementary Figure 3). Then, after penile hemodynamics stabilization (reaching a plateau), the pudendal nerve was stimulated for 30 sec to produce ICM isometric tetanic contractions with afterload, thus pushing some of the blood from the crura into the CC distal part, thereby sharply increasing ICP to a suprasystolic level of 209 ± 81 mm Hg (Figures 4 and Supplementary Figure 3).

In this case, the muscle’s work is defined as the difference in the potential energy accumulated in CC when ICP reaches a certain level. Assuming that the muscle tends to contract in an infinitely short time, forming an infinitely high blood flow and pushing a specific finite volume of blood *∆V* out of the crura, it can be described mathematically using the generalized Dirac function as follows: 



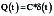

 (1)


where *Q(t)* is the blood flow rate, *C* is some constant, δ(t) is the Dirac delta function:



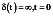





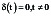

 (2)




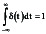



To determine the constant *C*, we will integrate the blood flow rate over time. It is known that the integral of blood flow rate over time is equal to the volume of blood passed over a specific time. 



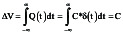

 (3)


Taking into account (3), the blood flow rate can be written as follows:



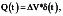

 (4)


The next step is to determine the power of muscle contraction:



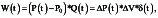



where P(t) -achievedICP,P_0_*-* ICPbeforethestartof muscle contraction.

To calculate the energy* E* accumulated due to the ICP increase, it is necessary to integrate the power over time:



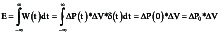

 (5)


Thus, the energy accumulated by CC and, consequently, the work performed by the muscle is equal to the product of the blood volume pushed from the crura and the amount of ICP change at the moment of muscle contraction.

In Figure 4A, due to muscle stimulation, ICP changed from 92 to 300 mm Hg. The total volume of blood pushed from the crura, according to performed cavernosometry, was 5 mL. In this case, given that 1 mm Hg ≈ 133 Pa, and 1 mL = 10^−6^ m^3^, we obtain:

*E* = 208*133*5*−10^−6^ ≈ 0.138 *J*

To prevent tunica albuginea deformation, muscles develop a force to counteract this stretch, which is approximately equal to this force but with the opposite vector. Hence, the tensile force *F* can be estimated as the product of the increase in pressure due to muscle contraction by the cross-sectional area of CC.



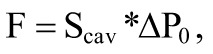

 (6)


**Example. Monkey #1 (** **Figure 4A** **).**

The cross-sectional area: *S_cav_* = 2.6 cm^2^. Pressure change:



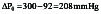





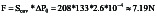



Thus, relying on the instrumentally measured parameters, we can calculate the contraction force of the pro-erectile muscles (ICM and BSM) with high accuracy.

In all experiments, we obtained an almost linear dependence of the ICP peak values (during pudendal nerve stimulation) on the initial ICP level (induced by cavernous nerve stimulation) (Figure 4B).

## Discussion

Erection involves intricate interactions among various physiological systems, including neurological, hormonal, vascular, and muscular components. While research on male sexual dysfunctions has primarily focused on hormonal, neurological, and vascular factors, there’s been a notable lack of exploration into the role of pelvic floor disorders in these dysfunctions. This stands in stark contrast to the considerable attention given to pelvic floor therapy in managing female sexual dysfunctions. Our study aimed to address this gap by analyzing the anatomy and physiological effects of pro-erectile muscles on penile hemodynamics during erection in olive baboons.

In this study, our objective was to investigate the anatomy and physiological effects of pro-erectile muscles on penile hemodynamics during the erection process in olive baboons. We focused on 3 key pro-erectile muscles: ICM, BSM, and LPM. Through our examination, we identified the anatomical characteristics of these muscles and their relationship with penile structures. Our findings revealed significant similarities between the muscle structure and function in baboons and humans. For example, the ICM, which tightly covers the penile crura and lower corpus cavernosum surface, displays contractile behavior similar to its human counterpart, contributing to penile rigidity during erection. Similarly, the BSM in baboons, resembling its human counterpart, facilitates the passage and release of semen during ejaculation by tightly covering the bulb and partially the corpus spongiosum. These similarities highlight the suitability of the olive baboon model for investigating penile anatomy and physiology, offering insights into human erectile function and potential therapeutic avenues. While there are some anatomical differences between baboons and humans, such as the larger size of muscles in baboons and the absence of LPM in humans, the fundamental functions and participation of these muscles in the erection process remain relatively similar.

We further conducted electrophysiological studies involving cavernous nerve and pudendal nerve stimulation to explore the influence of pelvic floor muscles on penile hemodynamics. Our analysis revealed several key findings:

1. A linear relationship between suprasystolic ICP values and ICP levels characterizes the system as closed from the moment of tetanic contraction of pro-erectile muscles.
2. The developed muscle power enables achieving full rigidity even at ICP levels lower than veno-occlusive values, suggesting a compression mechanism for deep veins collecting blood from the corpora cavernosa.
3. Studying muscle functional abilities in baboons expands therapeutic possibilities for treating certain types of erectile dysfunction through physical muscle training.
4. A quantitative assessment of muscle contribution to the erectile process in baboons lays the groundwork for applying this approach to humans as an objective criterion for evaluating the quality of medical care in treating erectile dysfunction.
5. The proposed mathematical algorithm, owing to its simplicity, can be utilized by urologists and andrologists to predict the treatment of erectile dysfunction caused by muscle dysfunction.

While our study utilized a non-human primate model, which closely resembles the human penile muscle system, there are minor anatomical differences that may affect muscle physiology. However, these differences are unlikely to significantly impact the conclusions or the proposed mathematical algorithm’s application in humans. Future human studies will provide further clarification and facilitate the algorithm’s successful integration into routine practice.

Additionally, a comprehensive examination of PFM training’s mechanistic aspects, particularly in comparison to PDE5 inhibitors, is warranted. Understanding the magnitude of pelvic floor muscle stimulation relative to PDE5 inhibitors could offer valuable insights into pelvic floor muscle training’s efficacy as a treatment option. Unlike PDE5 inhibitors, which are associated with side effects and limited efficacy in certain populations, pelvic floor muscle training may provide more sustainable benefits by addressing underlying muscular and vascular issues. Therefore, exploring the comparative benefits of pelvic floor muscle training and PDE5 inhibitors could enhance our understanding of pelvic floor muscle training’s potential as an alternative or complementary therapy for erectile dysfunction.

Overall, the data presented in our study, along with physiological data from previous research, contribute significantly to our understanding of the penile erection process and erectile dysfunction mechanisms.

## Figures and Tables

**Table 1.** Bulbospongiosus Muscle: Key Anatomical Findings

Part	Length	Origin	Direction	Insertion	Key Funcitons
Anterior part	3.8 ± 0.7 cm	CS TA median suture, over a length of 22.7 ± 2.3 mm	Oblique	Partially interwoven into the TA of the CC lateral surface Other fibers merge into the tendons and firmly attach to the TA of the CC dorsum	Interconnected tendons of BSM and ICM form a tendon loop providing robust support for the attached muscles and securing the anterior part of the bulb to the CC Compression of the CS from the sides promotes blood flow from the bulb into the CS distal, particularly into the glans
Middle part	2.7 ± 0.3 cm	Median suture in the bulb area, over a length of 2.8 ± 0.7 cm	Oblique	Cover the lateral surfaces of the bulb and intertwining with the TA of the CC, forming short tendons before attachment	Compression of the deep and urethral veins hinders venous return and increases pressure in the glans and CC
Posterior part	1.7 ± 0.3 cm	Central tendon of the perineum	Transverse	Interwoven into the posterior part of the bulb along the midline	Fixation of the bulb and its displacement toward the center of the perineum

BSM, Bulbospongiosus muscle; CC, Corpus cavernosum; CS, Corpus spongiosum; ICM, Ischiocavernosus muscles; TA, Tunica albuginea.

**Table 2.** Ischiocavernosus Muscles: Key Anatomical Findings

Length	Origin	Direction	Insertion	Muscle Parts	Function
4.5 ± 0.4 cm	Ischial tuberosity, ramus of the ischium, and sacrotuberous ligament (behind CC crura)	Longitudinal	Midway along its length, the ICM transitions into a tendon Fiber bundles that envelop the CC crura on 3 sides	Lateral part: attached to the lateral and dorsal surfaces of the CC. The tendon attaches to the periosteum of the pubic bone, forming a tendon bundle 8.1 ± 1 mm in width and 2 ± 0.3 mm in thickness. A broader bundle (width 13.5 ± 1.3 mm) is attached to the symphysis.	Press the CC crura against the pubic symphysis
				Middle part: the longest muscle fibers, with transition to tendon occurring only at the end. Fibers are interwoven into the lateral surfaces of the CC and cover 2.3 ± 0.3 cm of the outer aspect of the penis.	Tension to the TA and displacement of its surface layer during erection
				Short tendons attach the medial part of the ICM to the lower surface of the CC in the region of the urethral groove. These tendons are connected to the TA and the septum of the penis and are intertwined with the corresponding tendons on the opposite side.	Stabilization of the cavernous-spongy contact in the area of the urethral groove

BSM, Bulbospongiosus muscle; CC, Corpus cavernosum; CS, Corpus spongiosum; ICM, Ischiocavernosus muscles; TA, Tunica albuginea.

**Table 3.** Levator Penis Muscle: Key Anatomical Findings

Length	Origin	Direction	Insertion	Function
3.8 ± 1.1 cm	Periosteum of the pubic bone	Bilateral, longitudinal	Transitioning into a tendon cord, which merges with Buck’s fascia at the middle of the penis, with further distal attachment to the baculum on the dorsal plane	Displaces the skin and foreskin in the proximal direction during an erection and contributes to the curvature of the flexor side of the glans penis
