# Supplementary figures and images for: Pelvic Floor Muscle Anatomy and its Contribution to Penile Erection in Olive Baboons
Source: Urol Res Pract. 2024 May 1;50(3):173–80. doi: 10.5152/tud.2024.23020 (PMC11562810; doi:10.5152/tud.2024.23020)

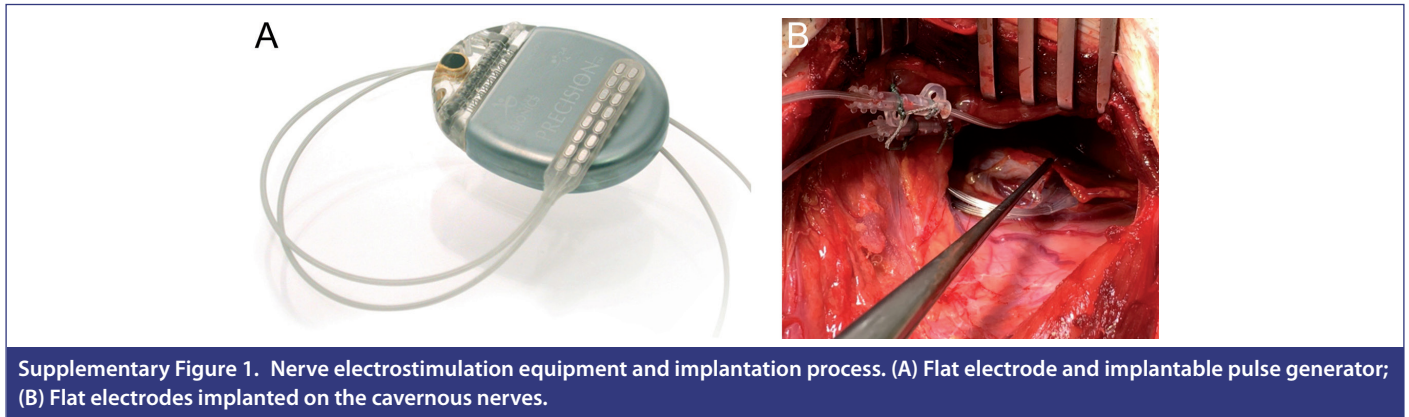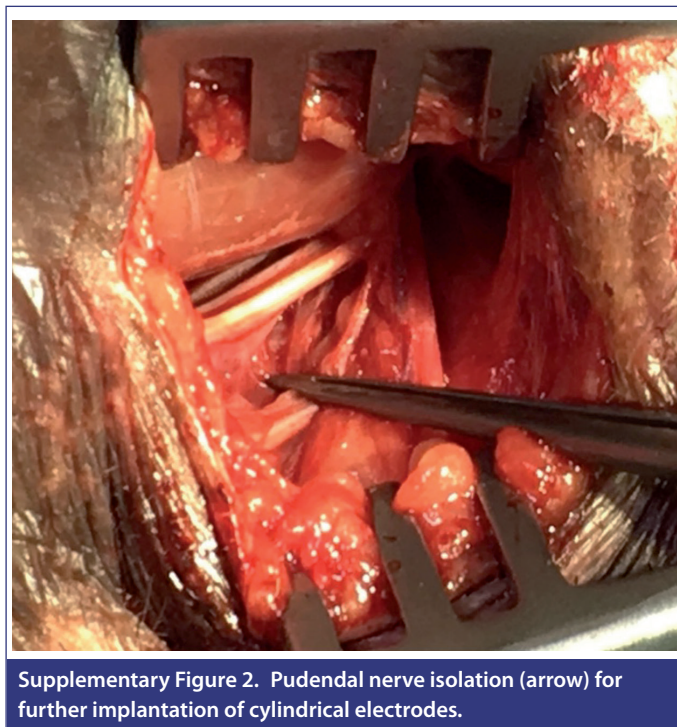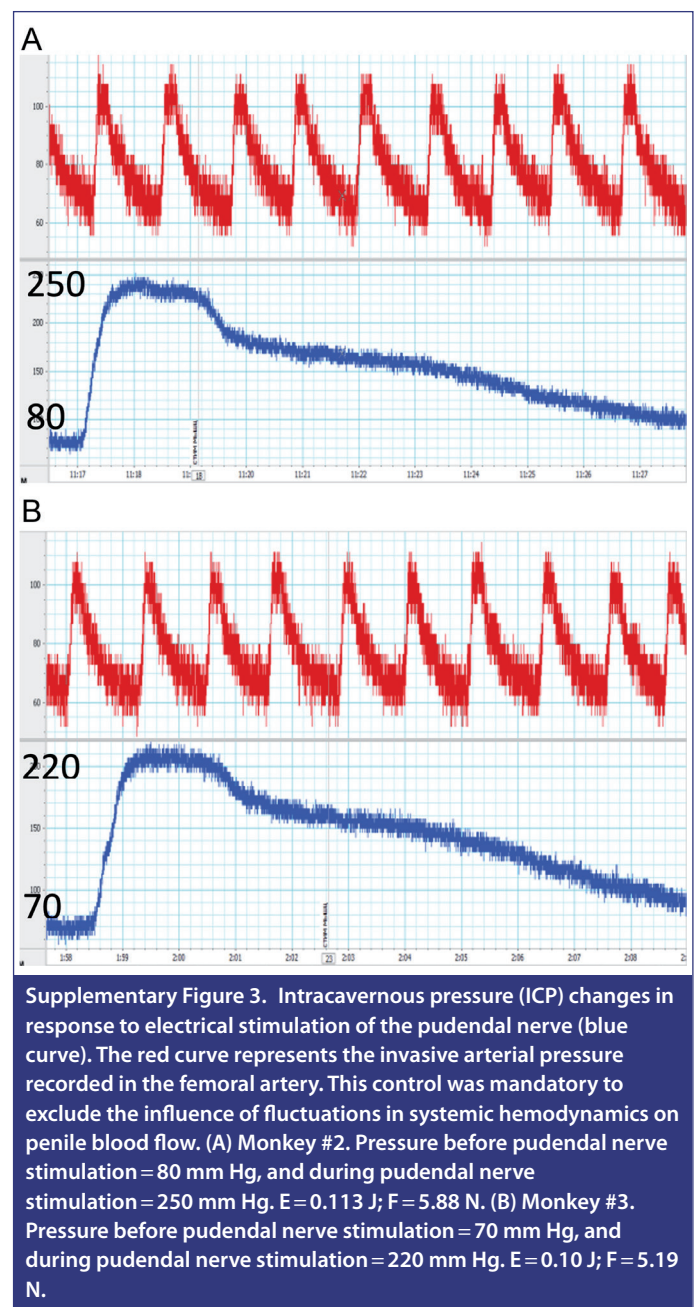

Supplement: Supplementary Material [file supplementary_material.pdf]
